# A Non-Synonymous Mutation in the Canine *Pkd1* Gene Is Associated with Autosomal Dominant Polycystic Kidney Disease in Bull Terriers

**DOI:** 10.1371/journal.pone.0022455

**Published:** 2011-07-27

**Authors:** Puya Gharahkhani, Caroline A. O'Leary, Myat Kyaw-Tanner, Richard A. Sturm, David L. Duffy

**Affiliations:** 1 School of Veterinary Science, The University of Queensland, Gatton, Queensland, Australia; 2 Centre for Companion Animal Health, The University of Queensland, Brisbane, Queensland, Australia; 3 Melanogenix Group, Institute for Molecular Bioscience, The University of Queensland, Brisbane, Queensland, Australia; 4 Epidemiology Group, Queensland Institute of Medical Research, Brisbane, Queensland, Australia; University Hospital Vall d'Hebron, Spain

## Abstract

Polycystic Kidney Disease is an autosomal dominant disease common in some lines of Bull Terriers (BTPKD). The disease is linked to the canine orthologue of human *PKD1* gene, *Pkd1*, located on CFA06, but no disease-associated mutation has been reported. This study sequenced genomic DNA from two Bull Terriers with BTPKD and two without the disease. A non-synonymous *G>A* transition mutation in exon 29 of *Pkd1* was identified. A TaqMan® SNP Genotyping Assay was designed and demonstrated the heterozygous detection of the mutation in 47 Bull Terriers with BTPKD, but not in 102 Bull Terriers over one year of age and without BTPKD. This missense mutation replaces a glutamic acid residue with a lysine residue in the predicted protein, Polycystin 1. This region of Polycystin 1 is highly conserved between species, and is located in the first cytoplasmic loop of the predicted protein structure, close to the PLAT domain and the second transmembrane region. Thus, this change could alter Polycystin 1 binding or localization. Analytic programs PolyPhen 2, Align GVGD and SIFT predict this mutation to be pathogenic. Thus, BTPKD is associated with a missense mutation in *Pkd1*, and the application of this mutation specific assay could reduce disease transmission by allowing diagnosis of disease in young animals prior to breeding.

## Introduction

Bull Terrier Polycystic Kidney Disease (BTPKD) is an autosomal dominant disease of English Bull Terriers which is characterized by bilateral renal cysts [1], [2] and leads to chronic renal failure in middle to old age [1]–[3]. Cysts can be detected using renal ultrasonography, the currently preferred method of diagnosis for BTPKD. However, this method is expensive and requires an experienced operator, and so a definitive diagnosis, especially early in the disease course, is difficult in some cases [1].

Polycystic Kidney Disease (PKD) has been reported in many species. However, English Bull Terriers, Persian cats, humans and rats suffer from a very similar disease in terms of mode of inheritance, age of onset, clinical signs and renal pathology [1], [2], [4]–[6]. Genetic diseases which are similar across species may be caused by mutations in orthologous genes [7].

While, the genetic cause of BTPKD is unknown. Studies in humans have shown that 85–90% of Autosomal Dominant Polycystic Kidney Disease (ADPKD) (OMIM ID: 173900) is caused by mutations in *PKD1* (Entrez Gene ID: 5310), with other cases caused by mutations in *PKD2* (Entrez Gene ID: 5311). Further, Autosomal Dominant Polycystic Kidney Disease in Persian cats (OMIA ID: 1451) is caused by a premature truncating mutation in the feline *Pkd1* orthologue (Entrez Gene ID: 100144606) [8].

In BTPKD, there is strong linkage between the BTPKD phenotype and the region near the canine *Pkd1* orthologue (Entrez Gene ID: 606755) on CFA06 [9]. However, no disease-associated mutations were identified in a previous study which sequenced *Pkd1* from cDNA made primarily from mRNA from peripheral blood [10]. This may have occurred because the *Pkd1* transcript is present in very low copy numbers, and the mutation was not detected. This is supported by the situation in humans and mice where *PKD1/pkd1* expression is greatest in fetal renal tissue, with low levels present in adult tissue [11], [12]. Alternately, the mutation may cause mRNA instability, also making detection of mutations difficult. Further, mutations leading to cryptic splicing, a deletion in part of *Pkd1*, and chromosomal duplications and rearrangements affecting *Pkd1*, were not excluded in the previous study. As only coding regions and the 3′ untranslated region (UTR) was sequenced, mutations in intragenic non-coding sequence and the 5′ UTR, non-exonic promoters, enhancers and other regulatory sequences may also not have been detected.

Thus, mutation detection studies on stable genetic material, genomic DNA (gDNA), would be useful to further investigate the role of *Pkd1* in BTPKD. This study aimed to screen gDNA from English Bull Terriers of known BTPKD status, for possible mutation/s associated with the disease. This study reports a mutation in *Pkd1* that is associated with BTPKD.

## Results

**Figure 1:**
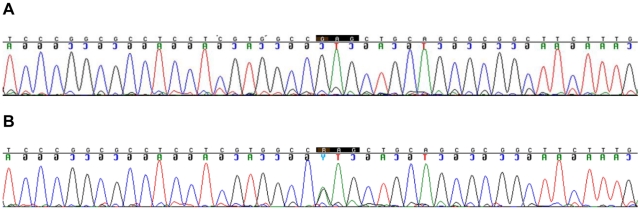
Chromatograms of the sequence in the region of the BTPKD-associated SNP in exon 29. (A) Chromatogram from an unaffected animal showing the animal was homozygous for the wild-type *G* allele. (B) Chromatogram from an affected animal showing the animal was heterozygous with *G/A* alleles. The changed DNA codon has been highlighted, and the nucleotide of interest is the first nucleotide of the codon.

Sequencing gDNA revealed a single non-synonymous *G* to *A* transition (a change in DNA codon *GAG* to *AAG*) (NCBI Assay ID: ss316885563) in cDNA nucleotide 9772 in predicted exon 29 of the canine orthologue of *Pkd1*. This sequence change was present in two BTPKD affected dogs but not in two unaffected dogs. Affected dogs were heterozygous for this sequence change (Figure 1).

### Sequence analysis

Thirty-seven allelic variants, including nucleotide substitutions, deletions and insertions, were identified in the 4 dogs whose *pkd1* gDNA was sequenced in this study. These variants were in the predicted 5′ UTR, 46 exons and 44 introns of *Pkd1* (Table 1). Of these 37 variants, the 9772G>A variant was the most likely to be disease-causing, as it was the only variant present in two BTPKD affected dogs but not in two unaffected dogs. Thirty six other variants were excluded from further analysis as they were less likely to be pathogenic as either; 1) all dogs were homozygous for the variant 2) all dogs were heterozygous for the variant 3) two normal animals and one animal with BTPKD were homozygous for the variant, and the other dog with BTPKD was heterozygous 4) one normal animal was heterozygous for the variant and the other dogs were homozygous for the wild-type allele 5) one dog with BTPKD was heterozygous for the variant and the other dogs were homozygous for the wild-type allele.

**Table 1:** Nucleotide variants and their location in canine *Pkd1* sequence.

Position/Haplotype	Nucleotide Number^1^	Nucleotide in public databases^2^	Dog 1 (affected)	Dog 2 (affected)	Dog 3 (unaffected)	Dog 4 (unaffected)
5′ UTR ss316885484^3^	171–192	*22A*	*22A/31A*	*22A/31A*	*22A/31A*	*22A/31A*
5′ UTR ss316885498	720	*T*	*T/C*	*C/C*	*C/C*	*C/C*
5′ UTR ss316885500	1326	*G*	*G/A*	*A/A*	*A/A*	*A/A*
Intron 4 ss316885502	234	*G or T*	*G/G*	*G/G*	*G/G*	*G/G*
Exon 7 ss316885504	122	*G*	*G/G*	*G/G*	*G/G*	*G/T*
Exon 7 ss316885506	206	*G*	*G/A*	*G/A*	*G/A*	*G/A*
Intron 7 ss316885508	110	*11C*	*10C/11C*	*10C/11C*	*10C/11C*	*10C/11C*
Intron 8 ss316885512	347	*A or G*	*A/G*	*G/G*	*G/G*	*G/G*
Intron 9 ss316885514	162	*A or G*	*A/G*	*G/G*	*G/G*	*G/G*
Intron 9 ss316885517	222	*A or G*	*A/G*	*G/G*	*G/G*	*G/G*
Intron 11 ss316885519	395	*T or G*	*T/T*	*T/T*	*T/T*	*T/T*
Exon 12 ss316885521	18	*A or G*	*A/G*	*G/G*	*G/G*	*G/G*
Intron 12 ss316885523	26–29	*ACC or GGG*	*GGG/GGG*	*GGG/GGG*	*GGG/GGG*	*GGG/GGG*
Intron 12 ss316885527	32	*A or G*	*G/G*	*G/G*	*G/G*	*G/G*
Intron 12 ss316885530	34–93	*59 bp present*	*Del/Del* 4	*Del/Del*	*Del/Del*	*Del/Del*
Intron 14 ss316885534	171–184	*11C or 13C*	*11C/13C*	*11C/13C*	*11C/13C*	*11C/13C*
Exon 15 ss316885536	1235	*C or G*	*C/G*	*C/C*	*C/C*	*C/C*
Exon 15 ss316885539	1693	*C or T*	*C/C*	*C/C*	*C/C*	*C/C*
Intron 16 ss316885541	275	*C or T*	*C/T*	*T/T*	*T/T*	*T/T*
Exon 17 ss316885543	85–86	*AA or GC*	*GC/GC*	*GC/GC*	*GC/GC*	*GC/GC*
Intron 17 ss316885546	41	*G or T*	*G/G*	*G/G*	*G/G*	*G/G*
Exon 23 ss316885548	125	*C*	*C/T*	*T/T*	*T/T*	*T/T*
Exon 23 ss316885550	518	*T*	*C/T*	*T/T*	*T/T*	*T/T*
Intron 23 ss316885552	239	*A*	*A/G*	*G/G*	*G/G*	*G/G*
Exon 26 ss316885554	5	*C or T*	*C/C*	*C/C*	*C/C*	*C/C*
Intron 26 ss316885556	33	*T or C*	*T/T*	*T/T*	*T/T*	*T/T*
Intron 26 ss316885559	926	*A or G*	*A/G*	*G/G*	*G/G*	*G/G*
Intron 27 ss316885561	63–74	*11C*	*10C/11C*	*10C/11C*	*10C/11C*	*10C/11C*
**Exon 29** 5 ss316885563	**42**	***G***	***G/A***	***G/A***	***G/G***	***G/G***
Intron 30 ss316885565	335	*C*	*C/T*	*T/T*	*T/T*	*T/T*
Intron 30 ss316885567	597	*C*	*C/T*	*T/T*	*T/T*	*T/T*
Exon 31 ss316885569	28	*G*	*T/T*	*T/T*	*T/T*	*T/T*
Intron 37 ss316885571	204	*C*	*C/T*	*T/T*	*T/T*	*T/T*
Intron 41 ss316885573	19	*6C*	*6C/7C*	*7C/7C*	*7C/7C*	*7C/7C*
Intron 41 ss316885575	117	*A or C*	*C/C*	*C/C*	*C/C*	*C/C*
Intron 42 ss316885578	52	*A*	*A/G*	*G/G*	*G/G*	*G/G*
Haplotype^6^			*Hap 5/Hap 4* 7	*Hap 5/Hap 2*	*Hap 1/Hap 2*	*Hap 1/Hap 3*

1 The position of each variant as the number of nucleotide residues from the 5′ end of the UTR, intron or exon.

2 Expected nucleotide residues based on the sequence available in the public databases. These nucleotides are considered as normal alleles, as they are not likely to cause the disease (refer to discussion). Where two nucleotides are recorded, there were two nucleotide variants reported for the relevant position in different databases.

3 NCBI Assay ID available at Single Nucleotide Polymorphism database (dbSNP).

4 Del; Deletion of 59 bp.

5 Mutation segregating with BTPKD. *G* is the wild type allele, and *A* the mutant allele.

6 Predicted haplotypes observed in the dogs (the nucleotide in bold indicates the BTPKD SNP):

Haplotype 1: 31A-C-A-G-G-G-10C-G-G-G-T-G-GGG-G-Del-11C-C-C-T-G-C-G-T-T-G-C-T-G-10C-**G**-T-T-T-T-7C-C-G.

haplotype 2: 22A-C-A-G-G-A-11C-G-G-G-T-G-GGG-G-Del-13C-C-C-T-G-C-G-T-T-G-C-T-G-11C-**G**-T-T-T-T-7C-C-G.

Haplotype 3: 22A-C-A-G-T-A-11C-G-G-G-T-G-GGG-G-Del-13C-C-C-T-G-C-G-T-T-G-C-T-G-11C-**G**-T-T-T-T-7C-C-G.

Haplotype 4: 22A-T-G-G-G-A-11C-A-A-A-T-A-GGG-G-Del-13C-G-C-C-G-C-G-C-C-A-C-T-A-11C-**G**-C-C-T-C-6C-C-A.

Haplotype 5: 31A-C-A-G-G-G-10C-G-G-G-T-G-GGG-G-Del-11C-C-C-T-G-C-G-T-T-G-C-T-G-10C-**A**-T-T-T-T-7C-C-G.

7 Hap; Haplotype.

Sequencing data was consistent with the presence of 5 haplotye blocks in the 4 dogs analyzed in this study (Table 1). Haplotype 1 was the background haplotype for the *G>A* mutation in BTPKD. Haplotypes 1 and 2 were the most frequent in these 4 dogs.

**Figure 2:**
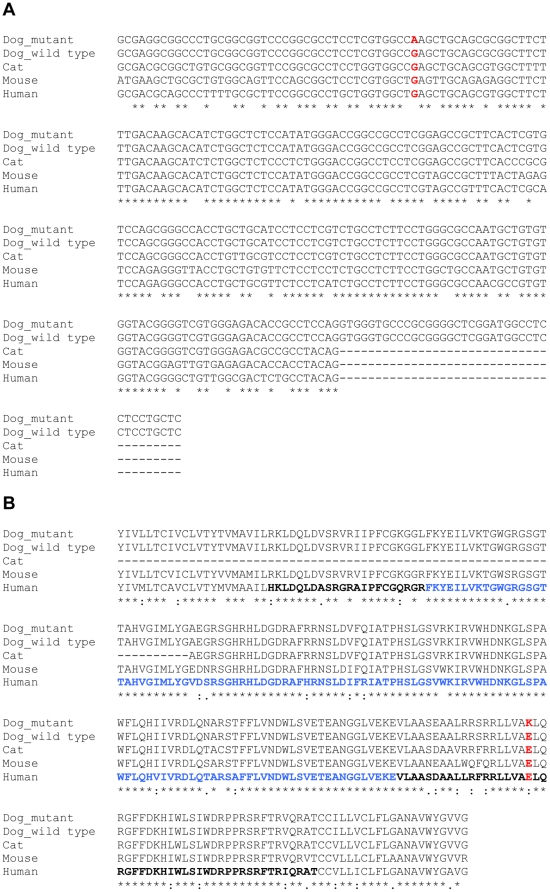
Nucleotide and amino acid sequence alignment in the region of the substitution. (A) Sequence of predicted exon 29 in mutant and wild type canine *Pkd1* has been aligned with that of cat, mouse and human. The mutation is highlighted in red. * Indicates conserved nucleotide residues. (B) Polycystin 1 has been aligned in the mutant and wild type dog, cat, mouse and human. The protein has been aligned from the beginning of the first transmembrane region (amino acid number 3081) to the end of the second transmembrane region (amino acid number 3309). The region with amino acids in bold is the predicted first cytoplasmic loop. The amino acids in blue bold indicate the predicted PLAT domain within the first cytoplasmic loop. The red bold represents the amino acid that predicted to have been changed in BTPKD. * Indicates conserved amino acid residues.

Alignment of predicted *Pkd1* exon 29 sequences in the dog, cat, mouse and human showed 74% homology, with all species having a G residue in the position of the BTPKD mutation or SNP (Figure 2).

### Genotyping

All 47 dogs with BTPKD had one copy of the *A* allele detected by the TaqMan® SNP Genotyping Assay for the BTPKD SNP. None of the 102 dogs without BTPKD had this SNP. Genotyping results were confirmed by sequencing a fragment containing the SNP in an additional 10 Bull Terriers. Results were consistent, with all five BTPKD affected dogs having the SNP but none of the five BTPKD unaffected dogs.

### Bioinformatic protein analysis

Bioinformatic protein analysis suggested the BTPKD-associated mutation would change an amino acid in the predicted canine Polycystin 1 protein (NCBI Accession Number: NP_001006651), in a conserved region close to the predicted PLAT (Polycystin 1, Lipoxygenase, Alpha-Toxin) domain and second transmembrane region. The amino acid change, E3258K, would change negatively charged glutamic acid to positively charged lysine. Figure 2, shows these regions are conserved between different mammalian species, with all species having a glutamic residue in the BTPKD-associated mutation location. There was 84% predicted homology between these species' amino acid sequence in the predicted first cytoplasmic loop of the protein, and 89% in the region of the PLAT domain.

PolyPhen-2, Align GVGD and SIFT were used to predict the effect of the amino acid substitution on the predicted encoded protein (Table 2). PolyPhen-2 classified this mutation as “probably damaging” with a 0.991/1 score. This score indicates the mutation is predicted to affect protein function or structure with a high degree of confidence.

**Table 2:** Prediction of pathogenicity of the predicted amino acid change in BTPKD as determined by PolyPhen-2, Align GVGD and SIFT.

Prediction Tool	Prediction data	
**PolyPhen-2**	Score	0.991
	Sensitivity	0.60
	Specificity	0.96
	Prediction	Probably damaging substitution^1^
**Align GVGD**	GV	0.00
	GD	56.87
	Prediction	Damaging substitution/Class C55^2^
**SIFT**	Sequences at position^3^	10
	Median sequence conservation	3.45
	Score^4^	0.00
	Prediction	Affects protein function

1 Probably damaging, i.e., it is predicted to affect protein function or structure with a high degree of confidence.

2 Substitution classification in Align GVGD: GD> = 55+Tan(10)x(GV∧2.0) = >Class C55.

3 Number of aligned sequences at the position of the substitution.

4 SIFT scores range from 0 to 1. The amino acid substitution is predicted to be damaging if the score is < = 0.05, and tolerated if the score is >0.05.

Align GVGD classified the BTPKD substitution as a class C55 change, with a GV score of 0 and GD score of 56.87, and a risk estimate of 2 to 2.5 for this change having a pathogenic effect on the protein (Table 2). This class of sequence changes is the second highest pathogenicity category, of out of the seven classes in the Align GVGD classification system [13].

SIFT also predicted this substitution could affect the protein function (score 0) with this assessment based on an alignment of Polycystin 1 predicted sequences from the dog, cat, human, Rhesus monkey, common chimpanzee, mouse, rat, horse, chicken, and Japanese medaka fish (Japanese rice fish). Amino acid substitutions with scores less than 0.05 are predicted to be deleterious, and those with scores greater than or equal to 0.05 are predicted to be tolerated.

## Discussion

This study has found a nucleotide transition in predicted exon 29 of the canine *Pkd1* orthologue that is highly likely to cause BTPKD. In this study of 149 dogs, the mutation was only present in 47 affected animals, and likely to be pathogenic based on the protein bioinformatic evidence. All BTPKD dogs were heterozygous for this sequence change, consistent with the findings in mice and cats that animals homozygous for *Pkd1* mutations die embryonically [14]–[16].

In this study more than 27 kb of the canine *Pkd1* orthologue was sequenced, however some regions were excluded (Figure S1). Only the 399 bp at the 3′ end of intron 1 was sequenced, as the rest of the predicted intron is a GC-rich area of more than 14 kb, and the sequence of the 5′ splicing site was unknown at the time of the study. Similarly, a region of 600 bp in predicted intron 30 was excluded from sequencing as the sequence was unknown at the time of the study. Finally, 79 bp of sequence in 5′ untranslated region extending from nucleotides 903 to 981 could not be sequenced, possibly due to the formation of hairpin or other secondary structures.

Thirty-six other nucleotide variants were found in canine *Pkd1*. However, these variants were less likely to cause the disease, not only due to inconsistencies with the mode of inheritance of BTPKD, but also 19 of these variants were present in sequence in public databases where, for example, NCBI sequence contained one allele and Ensembl a second. These database sequences were considered likely to be non-disease-associated variants as *Pkd1* sequence in Ensembl is from Boxer gDNA, a breed not reported with PKD. Similarly, the sequence in NCBI is from Madin-Darby Canine Kidney Epithelial Cells (MDCK) from a Cocker Spaniel, another breed not reported with PKD. However, some of these variants could still play a role in disease, and further studies could investigate this possibility.

The BTPKD mutation was not detected in a previous study which sequenced *Pkd1* from mRNA extracted from white blood cells of BTPKD affected and normal animals [10]. The samples used in the current study were from the same animals used in this previous study, suggesting the mutant mRNA may be unstable and degrade rapidly [17]. Expression analysis, using methods such as quantitative PCR, to measure Polycystin 1 expression in affected tissues would have been useful to investigate this. However, suitable kidney tissue samples were not available for such analysis.

The exon 29 SNP that segregated with the BTPKD phenotype is predicted to result in an amino acid change in the predicted protein, Polycystin 1, replacing a negatively charged glutamic acid residue with a positively charged lysine residue. Charge reversal amino acid substitutions may have deleterious effects on proteins. While the structure and domains of canine Polycystin 1 have not been studied, the comparable amino acid is located within the first cytoplasmic loop of human Polycystin 1 (NCBI Accession Number: NP_001009944), 19 amino acids downstream from the PLAT domain, and 31 amino acids upstream of the second transmembrane region. These regions are highly conserved between different mammal species.

The role of the intracellular and extracellular loops between the transmembrane domains of the protein is still unknown, but Polycystin 1 is known to bind to both focal adhesion and cell-cell adhesion proteins, forming a Polycystin 1 complex [18]–[21]. Focal adhesion sites are important in linking the extracellular matrix and the actin cytoskeleton, regulating cell-matrix interactions, motility, and signal transduction [21]–[25]. As the BTPKD predicted amino acid change is located close to the PLAT domain and transmembrane region, this change may alter cell-matrix interaction, mobility or signal transduction of the protein. Further, being close to the transmembrane region of the protein, the BTPKD predicted amino acid change could alter localization/trafficking of the protein. Such altered protein trafficking has been suggested to occur in mutant Polycystin 1 in ADPKD and PKD mice models, as well as in other inherited diseases in humans involving immunoglobulin-like proteins [14], [26], [27]. Further studies are being conducted to investigate the effect of this mutation on the cellular location and function of Polycystin 1 by expressing both the wild type and the mutant proteins in Madin-Darby Canine Kidney (MDCK) cells.

Identification of the mutation that is likely to cause BTPKD supports the first cytoplasmic loop of Polycystin 1 being important in the protein's function. Little is known about this protein region in humans or other species, and further studies on the effect of this mutation on the structure and function of Polycystin 1 could increase understanding about the protein.

Studies in ADPKD and PKD mice models have shown that Polycystin 1 is present in high levels in fetal kidney tissues, but only in low levels in adult tissue [11], [12]. While cystogenesis does occur in some cases *in utero*, a second somatic *PKD1/Pkd1* mutation appears to be required in a cell for a cyst to form from clonal expansion of this cell [14], [15], [28], [29], and as at least some humans with ADPKD develop cysts in adulthood [30], [31], it is likely that the mutation continues to have a clinically significant effect beyond the fetal stage. Other factors beside age of the patient are also likely to play a role in cystogenesis including level of Polycystin 1 expression, and penetrance of pathogenic alleles [15], [32]–[34].

The pathogenicity prediction tools, PolyPhen-2, Align GVGD and SIFT also supported this amino acid substitution being pathogenic. PolyPhen-2 is a software tool that predicts the possible impact of amino acid substitutions on the structure and function of proteins using biophysical and evolutionary comparisons. Align GVGD is a program that combines the analysis of the biophysical characteristics of amino acids and protein multiple sequence alignments to predict the effect of mutations on the encoded proteins. Finally, SIFT produces predictions about mutation pathogenicity using amino acid sequences which are conserved among different species, using multiple sequence alignments. The prediction results obtained using these tools are in agreement with each other, and suggest that this mutation is in a conserved region of Polycystin 1 and has a high potential to affect the structure and function of the protein.

Further support for the pathogenicity of the *Pkd1* SNP found in the BTPKD-affected dogs in this study comes form studies in other species. In humans, mutations in *PKD1* are responsible for up to 85–90% of ADPKD cases. Mutations are found anywhere in the gene, and include nonsense, missense, frame shift, splicing, deletions and insertions (http://www.hgmd.cf.ac.uk/ac/index.php). Around 50% of the mutations found in *PKD1* in humans are classified as missense/nonsense mutations, and around half of these are missense mutations. While the missense mutation associated with BTPKD in this study has not been reported in ADPKD, 10 missense mutations in *PKD1* producing amino acid changes in the first cytoplasmic loop of the protein have been reported [34]–[40], of which six were shown to segregate with the disease phenotype [35], [38], [40]. The closest of these missense mutations to the BTPKD SNP is a R3247H mutation, five amino acids upstream of the equivalent position of the BTPKD mutation in human Polycystin 1. This mutation produces a conservative substitution of arginine to histidine.

Three further disease-related glutamic acid to lysine changes have been reported in ADPKD [34], [36], [38], [40]. One of these E to K changes in ADPKD in the *PKD1* gene is an E3604K mutation that has been classified as likely to be pathogenic [38]. Rossetti *et al* (2001) have also reported that a putative missense mutation in position 2771 segregated with the disease in four unrelated families and was not observed in 230 normal cases, supporting its role in the disease. Rossetti *et al* (2002) have also reported the same amino acid change at amino acid position 1811 in ADPKD patients. While, they were not able to test whether this change segregated with the disease due to lack of samples [40], this amino acid change has also been reported to be pathogenic in other proteins as it changes their function [41], [42]. Thus, mutations which are similar to that in BTPKD are pathogenic and so also likely to be disease causing in ADPKD.

Interestingly, feline PKD is due to *C>A* transversion in predicted exon 29 of feline *Pkd1*, resulting in a stop codon at the equivalent position of 3284 in human Polycystin 1 [8]. This mutation is located in the equivalent region to the first transmembrane domain of human Polycystin 1, only 32 amino acids downstream of the BTPKD mutation. Feline PKD is another similar disease to ADPKD and BTPKD, providing further support for the BTPKD-associated mutation's causative role in the disease.

Development of the TaqMan® SNP Genotyping Assay is a useful molecular genetic diagnostic test to diagnose BTPKD. This test is favoured for detecting single nucleotide polymorphisms in large-scale population mutation screening, as it is accurate, sensitive, specific, rapid and cost effective [43]. Such a diagnostic test would allow breeders to diagnose disease prior to breeding, and thus prevent breeding of affected animals. It would also allow the diagnosis of animals prior to development of clinical disease, and so aid early treatment. Currently, ultrasonography is the preferred method of diagnosis for BTPKD, as it is sensitive, noninvasive, and quick, if performed by a highly skilled operator [30], [31]. However it is costly and the presence of cysts is nonspecific for BTPKD. Ultrasonography may also not detect disease in dogs with late enlargement of renal cysts [1]. In addition, where less than 3 cysts are detected, or the cysts are present only in one kidney, definitive diagnosis requires retesting, pedigree inspection and possibly test mating. Moreover, variation in the echogenicity of normal kidneys in Bull Terriers can also make it difficult for ultrasonography to give a definitive diagnosis [1]. Thus, this accurate, easy and inexpensive molecular genetic diagnostic test will be useful to detect BTPKD at any age, from a sample of blood, hair or a buccal swab.

All cases of reported BTPKD to date in Australia descend from two affected half-siblings, making it very likely that there is only one disease-causing mutation in the Australian Bull Terrier population. Interestingly, these half-sibs were recently descended from an imported animal, suggesting the disease may well be international and due to this one mutation. Thus, a mutation-based diagnostic test is desirable in this disease, as these tests are accurate, sensitive, specific and inexpensive. They also avoid some of the inaccuracies of a linkage-based diagnostic test, which detect and analyze the presence of a genetic marker located near the disease locus. Despite this, due to differences in the clinical presentation, inheritance and pathology of PKD in other canine breeds, it is unlikely the same mutation is the cause of PKD in the Cairn Terrier [44] or West Highland White Terrier [45].

In summary, a *G>A* transition at nucleotide 9772 in cDNA in predicted exon 29 of canine *Pkd1* segregates with the BTPKD phenotype and results in a predicted change of a glutamic acid to a lysine residue at amino acid number 3258 of the predicted canine Polycystin 1 protein. A TaqMan® SNP Genotyping Assay has been developed to identify this mutation.

## Materials and Methods

### Ethics Statement

The University of Queensland, Animal Ethics Committee, has approved this study.

### Selection of dogs and DNA extraction

Diagnosis of BTPKD was established using ultrasound as described previously [1]. Unaffected Bull Terriers were over one year of age. Genomic DNA from 47 affected and 102 unaffected Bull Terriers was extracted from peripheral blood using a salting-out extraction method [46]. All affected dogs were descended from two half-siblings. The *Pkd1* orthologue was sequenced from gDNA from four closely related animals; two affected with BTPKD and two unaffected.

### Amplification of *Pkd1*

Canine *Pkd1* sequence was obtained from databases NCBI (http://www.ncbi.nlm.nih.gov/), and Ensembl (http://ensembl.genomics.org.cn/Canis_familiaris/Info/Index). At the time of the study, the gene sequence was integrated into the canine genome map with the exception of missing sequence from predicted introns 1 and 30.

For PCR amplification canine *Pkd1* was divided into 22 fragments 500–2000 bp in size (Table S1). Intron 1 was not amplified as it was 14 kb, GC-rich and the 5′ sequence was unknown. However, 399 bp at the 3′ end of this intron was amplified to investigate 3′ splicing sites for intron 1. Primers were designed in predicted exonic sequence where possible using Primer 3 version 0.4.0 (http://frodo.wi.mit.edu), and analyzed using OligoAnalyzer version 3.1 (http://www.idtdna.com/analyzer/applications/oligoanalyzer/) (Table S1).

Fragments were amplified in polymerase chain reactions (PCR)s containing 1× PCR Buffer containing 0.3 mM dNTPs and 1.5–2.5 mM MgSO_4_ (QIAGEN, Hilden, Germany), 0.5–1 µM forward and reverse primers, 0.5 U HotStar HiFidelity DNA Polymerase (QIAGEN, Hilden, Germany), 0–1× Q solution, and 10–40 ng DNA in a 10 µl reaction volume. Standard thermocycling conditions were 95°C for 5 min, followed by 35 cycles of 94°C for 30 sec, 60°C for 1 min, and 72°C for 2 min; and one cycle of 72°C for 10 mins (Table S1).

To confirm the size of the PCR products, samples were stained using Blue/Orange 6× loading dye (Progema, Madison, USA) and electrophorezed (BIO-RAD, Hercules, USA) on a 1.5% agarose gel (Progema, Madison, USA) using 1× TAE Buffer (Promega, Madison, USA), 10000× SYBR® Safe DNA gel stain (Invitrogen Eugene, Oregon, USA) and size standard Lambda DNA/EcoR+HindIII (Promega, Madison, USA), and visualized using a transilluminator (BIO-RAD, Hercules, USA).

### Sequencing of *Pkd1*

PCR products were purified using MinElute PCR Purification Kit (QIAGEN, Hilden, Germany) and sequenced bidirectionally using forward, reverse and internal sequencing primers. The standard 20 µl sequencing reaction contained 10–100 ng purified DNA, 1 µl Big Dye Terminator version 3.1 (Applied Biosystems, Foster City, USA), 3.5 µL BigDye Sequencing Buffer (Applied Biosystems, Foster City, USA) and 3.2 pmol sequencing primer. Standard sequencing conditions were 94°C for 5 mins, followed by 30 cycles of 94°C for 10 sec, 50°C for 30 sec, and 60°C for 2 mins (Table S1). Unincorporated dye terminators were removed using an ethanol/EDTA/sodium acetate precipitation method, according to the BigDye® Terminator v3.1 Cycle Sequencing Kit protocol (Applied Biosystems, Foster City, USA), the pellet diluted and run on a 3130X/Genetic Analyzer (Applied Biosystems, Foster City, USA).

### Sequence analysis

Sequences were analyzed and assembled using ChromasPro, version 1.33 (Technelysium Pty Ltd, Queensland, Australia) and Sequencher, version 4.7 (Gene Codes Corporation, Ann Arbor, USA). Consensus sequence was compared with those in public databases NCBI http://blast.ncbi.nlm.nih.gov/Blast.cgi, and Ensembl http://www.ensembl.org/Multi/blastview and from BTPKD affected and unaffected animals.

### Bioinformatic protein analysis

The translate tool http://au.expasy.org/tools/dna.html from the ExPASy Proteomics Server was used to predict the change in amino acid residue caused by the mutation. In order to predict the location and effect of the amino acid change on the predicted canine Polycystin 1 structure, it was compared to the well-annotated human Polycystin 1. Thus, the predicted sequence of canine Polycystin 1 in normal dogs and BTPKD dogs was compared with that of Polycystin 1 from humans by using the following databases and tools: UniProt http://www.uniprot.org from European Bioinformatics Institute (EMBL-EBI), Protein http://www.ncbi.nlm.nih.gov/protein and Conserved Domain Database http://www.ncbi.nlm.nih.gov/Structure/cdd/cdd.shtml from National Centre for Biotechnology Information (NCBI). ClustalW2 http://www.ebi.ac.uk/Tools/clustalw2/index.html from EMBL-EBI and COBALT http://www.ncbi.nlm.nih.gov/tools/cobalt/ from NCBI were used to align canine Polycystin 1 sequence with orthologous sequence in other species. Tools including PolyPhen-2 http://genetics.bwh.harvard.edu/pph2/dokuwiki/start, Align GVGD http://agvgd.iarc.fr/agvgd_input.php and SIFT http://sift.jcvi.org/ were used to predict the affect of the predicted altered amino acid on the encoded protein.

### Genotyping

The BTPKD-associated SNP was investigated using TaqMan® SNP Genotyping Assay (Applied Biosystems, Foster City, USA) in a total of 47 affected and 102 unaffected Bull Terriers from a large pedigree in which BTPKD was segregating. All the animals were more than one year old at the time of the diagnosis to ensure the diagnosis was correct [1]. A Custom TaqMan® SNP Genotyping Assay (Applied Biosystems, Foster City, USA) using specific PCR primers (sequence of forward primer TCTGTCCGTCCGTCCCT, and reverse primer GCCAGATGTGCTTGTCAAAGAAG), and probes (probe sequence to detect the wild type allele was TGGCCGAGCTGCAG labelled with VIC® fluorescent dye, and probe sequence to detect the mutant allele was TGGCCAAGCTGCAG labelled with FAM™ fluorescent dye). Final concentrations of 1× TaqMan® SNP Genotyping Assay Mix, 1× TaqMan® Genotyping Master Mix, and 10 ng canine gDNA were mixed in a 25 µl total volume on a 96-well plate, and the assay performed using a 7500 Real Time PCR System (Applied Biosystems, Foster City, USA). The results were analyzed using 7500 Software Version 2.0.4 (Applied Biosystems, Foster City, USA). Fragment 19 was amplified and sequenced from gDNA from five Bull Terriers with BTPKD and five Bull Terriers without BTPKD (Table S1), to confirm SNP genotyping and sequencing results.

## Supporting Information

Figure S1 **Canine** ***Pkd1*** **gene.** This figure shows the canine *Pkd1* gene from the 5′ untranslated region (UTR) to the 3′ untranslated region. The solid boxes represent the 46 predicted exons. The regions in 5′ UTR, intron 1 and intron 30 marked with clear ovals are regions of the gene that were not sequenced in this study.
(TIFF)

Table S1 **Conditions used for amplification and sequencing of Canine** ***Pkd1*** **gene.** This table shows sequences of primers, and PCR amplification and sequencing reaction conditions used for sequencing canine *Pkd1* from genomic DNA.
(DOC)
