# Supplementary figures and images for: A Non-Synonymous Mutation in the Canine Pkd1 Gene Is Associated with Autosomal Dominant Polycystic Kidney Disease in Bull Terriers
Source: PLoS One. 2011 Jul 27;6(7):e22455. doi: 10.1371/journal.pone.0022455 (PMC3144903; doi:10.1371/journal.pone.0022455)

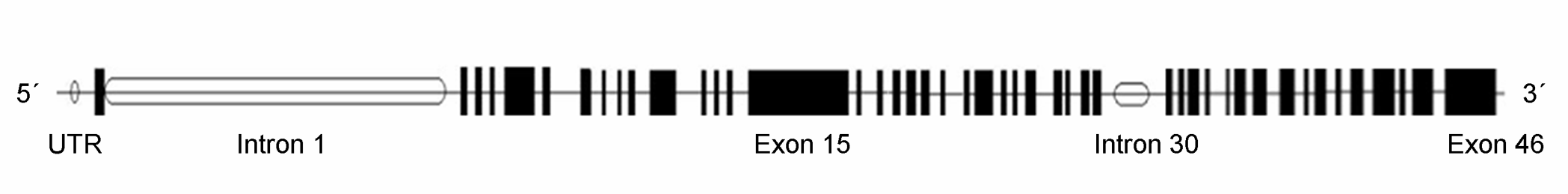

Supplement: Figure S1 — Canine Pkd1 gene. This figure shows the canine Pkd1 gene from the 5′ untranslated region (UTR) to the 3′ untranslated region. The solid boxes represent the 46 predicted exons. The regions in 5′ UTR, intron 1 and intron 30 marked with clear ovals are regions of the gene that were not sequenced in this study. (TIFF) [file pone.0022455.s001.tif]
