# Supplementary material for: A Non-Synonymous Mutation in the Canine Pkd1 Gene Is Associated with Autosomal Dominant Polycystic Kidney Disease in Bull Terriers
Source: PLoS One. 2011 Jul 27;6(7):e22455. doi: 10.1371/journal.pone.0022455 (PMC3144903; doi:10.1371/journal.pone.0022455)
Supplement: Table S1 — Conditions used for amplification and sequencing of Canine Pkd1 gene. This table shows sequences of primers, and PCR amplification and sequencing reaction conditions used for sequencing canine Pkd1 from genomic DNA. (DOC) [file pone.0022455.s002.doc]

| Fragment | Start^1^ | End^1^ | PCR primers | Sequencing primers | Variations from standard conditions in amplification PCRs^5^ | Variations from standard condition in sequencing PCRs^6^ |
| --- | --- | --- | --- | --- | --- | --- |
| 1 | 1711 bp^2^ 5’ to start codon | 787 bp 5’ to start codon | First round:  F^3^:  ATCCTCCCTCAGCCCTAAGAC  R^4^:  GTCCGAGCGTCCGCAGTC  Second round  F:  TGAGCACAGTGAGGGATCTG  R:  CGGGGAATGGATAACTGCTA | F2: TTAGGCATCCATCCACCAG  F3: GTGTGATCCTGGAGACCTGG  R2: CCAGGTCTCCAGGATCACAC  R3: GGATCCCCTGCCCTGTAAATTG | First rounds:  62°C, 35 cycles  Second round:  62°C, 45 cycles | Initial denaturation of 98°C for 10 min, PCR cyclical denaturation of 98°C for 30 sec, annealing temperature of 60°C |
| 2 | 926 bp 5’ to start codon | 181 bp inside Exon 1 | F:  TCACCCTTTTAAGAGCCCAAT  R:  GTCCGAGCGTCCGCAGTC | F2: AGTCGGGAGCCTGAGGAC  F3: GGTCATCACCGCTAGTGCTTG  R2: CCTGTCTGCTCGCGCTAC  R3: AGCGCGCTCGCGCTGCAG | 58°C, 45 cycles | For sequencing using reverse primer: initial denaturation of 98°C for 10 min, PCR cyclical denaturation of 98°C for 30 sec, annealing temperature of 60°C |
| 3 | 399 bp 5’ to Exon 2 | 73 bp inside Exon 4 | F:  CTCTTCCCTGTGGCCTGTC  R:  CAGGCCGAACCACGTGAAC | R2: GTACCCGGACAAGCACAGC | 60°C, 35 cycles | N/A^7^ |
| 4 | 215 bp inside Intron 3 | 54 bp inside Exon 6 | F:  AACCATCTGCCCTGCCGTC  R: TACCAGGGAAGATGACGGTG | F2: CCTGCCTCCCTGACAACAGC  R2: CCATCACCGAAGTCCCAG | 60°C, 35 cycles | N/A |
| 5 | 598 bp inside Exon 5 | 180 bp inside Exon 7 | F:  AGACCCTCAAGTTTGGCATC  R:  CACAGGTCCGTGTTGCACT | F2: CATCCAGCACTTCCTGGTCTC  R2: GGCAACTCTGGGTGTAGC | 65°C, 35 cycles | N/A |
| 6 | 78 bp inside Exon 7 | 43 bp inside Intron 11 | First round:  F:  GGGAGGGTGCTGTCTGTG  R:  GCAGGCGTAGGGCTTCTG  Second round:  F:  CCTCGAGAGCTGCCAGAAC  R:  CTGAGGCTGTGGTGCTAACC | F2: GGGCTCCTTCCTTAGCTGTTC  F3: CCTCCACCACGCAGACAG  F4: GTCCCATGGCTACTGGATTG  R2: GGTACGCTGGTCTTGCTGATG  R3:  TCCCACGAGGAAGTTCTCCG | First round:  60°C, 35 cycles  Second round: 62°C, 20 cycles | N/A |
| 7 | 114 bp inside Exon 10 | 650 bp inside Exon 11 | F: CCAGAGCCAGTATCCACCTGC  R: CTGTTCTCCACCATGATCTCC | F2: CTGAGCCTGTGCCACTGTCC  R2: GGTGGTGAGGCGCTCTGTAG  R3: GCAGGCGTAGGGCTTCTG | 60°C, 35 cycles | N/A |
| 8 | 243 bp inside Exon 11 | 119 bp inside Exon 13 | First round:  F:  CTACAGAGCGCCTCACCACC  R: TTGTATGGAGGCTTGAACTGG  Second round  F:  CTGGGCGCTATGAGGTCC  R:  GTGTTGGGAGGCAACACG | F2: GGAGATCATGGTGGAGAACAG  F3: AAGGGCCGTCTCTAGCTGC  R2: CTGGTAGATGACGTTGAAGACC  R3: TGAGTTGTGGACAGCACAGG | First round:  60°C, 35 cycles  Second round:  60°C, 28 cycles | As for fragment 1 |
| 9 | 63 bp inside Exon 12 | 508 bp inside Exon 15 | First round:  F:  AGGTACAGCCCCGTGGTAG  R:  CGAACGTCCAGTCAAAGATG  Second round  F:  GCTGGACCATTGATGACAAG  R:  TGTGCCCGCAGGTAAACG | F2: CTCCAACCACGTGAGCAAC  F3: CTGGGTCCAAGAGCATTGTC  R2: ACTTGCTGCGTCAGGTTCTC  R3: CAACCCAGACCAGATGGAGG | First round:  60°C, 35 cycles  Second round:  62°C, 28cyles | As for fragment 1 |
| 10 | 374 bp inside Exon 15 | 1795 bp inside Exon 15 | F:  AGCCTGAGCCCATCTGTG  R:  AGTTGGACGTGGTATGTG | F2: CATCTTTGACTGGACGTTCG  R2: CGGAAGGTGTAGGAGATGG | 60°C, 35 cycles | N/A |
| 11 | 1337 bp inside Exon 15 | 2910 bp inside Exon 15 | F:  CGGCACCAACATCTCCTAC  R:  ACACCTGCCAGCGGTACTCG | F2: GGTCAGTGGCCTCAGCATCC  R2: CCGAAAGGATGACCAGTGAG | 60°C, 35 cycles | N/A |
| 12 | 2744 bp inside Exon 15 | 548 bp inside Intron 16 | F:  ACCTCGTGAGCTTCTTCGTG  R:  CCACCTCAGCCAATCCAGG | N/A | 60°C, 35 cycles | As for fragment 1 |
| 13 | 359 1bp inside Exon 15 | 133 bp inside Exon 17 | F:  CAGACACCACTCAGCTTCC  R:  GAACCCTCGCTGCAGTTATC | N/A | 60°C, 35 cycles | As for fragment 1 |
| 14 | 45 bp inside Exon 17 | 64 bp inside Exon 20 | F:  CATCGTGTCCTTGGAGTGC  R:  GGTGAGGTCTGTGGGTCC | N/A | 62°C, 35 cycles | As for fragment 1 |
| 15 | 125 bp inside Exon 19 | 135 bp inside Exon 21 | F:  TCTCTGCCTACGGAGCTGTG  R:  GCCGCGATCTGCTGGATGTC | N/A | 62°C, 35 cycles | As for fragment 1 |
| 16 | 38 bp inside Exon 21 | 537 bp inside Exon 23 | F:  GTGGTGGCAGAGGAGGATG  R:  CCTGGGGCTGGATGACTG | F2: GGTCCAGGTTGAGCCTAGTC  R2: CCTGGATGCTACCATGAGTG  R3: CCCTGGACAGTGGAGTGG | 60°C, 35 cycles | N/A |
| 17 | 321 bp inside Exon 23 | The last nucleotide of Exon 24 | F:  TGTGGTGCAGCTCATGTTCC  R:  CCTGGGGCAATGAAGAAG | N/A | 60°C, 35 cycles | N/A |
| 18 | 35 bp inside Exon 24 | 119 bp inside Exon 27 | F:  GAGCCTGAGCCCTACCTG  R:  GGTGGCGATCTGGAACACG | F2: ATCTGACCAGCCACTTCCAC  F3: TTCAAGTATGAGATCCTGGTCAAG  R2: CGGTGATAAACACACGCAACG  R3: CTGCTCGGCAACCACTGTCC | 62°C, 35 cycles | N/A |
| 19 | 29 bp inside Exon 27 | 877 bp inside Intron 30 | F:  CACACGTGGGCATCATGCTG  R: CAGCAGGGTGAAGTCAGTAGG | F2: GGTGTCCAGTGTGGTCGTC  F3: GGAGCAGCGATCTAAGACCT  R2: CTGACTCTCCCGCTCTGC  R3: CCCGTACCACACAGCATTG | 60°C, 35 cycles | N/A |
| 20 | 1494 bp Inside Intron 30 | 115 bp inside Exon 36 | F:  ACCTCGCCCTCCACTGAC  R: GAGCCTACCCAGCCAGAGAC | F2: TGCTGGACAAATGAAAAGCG  F3: GTCCATCATGGGCAGCA  R2: TCAGCGTTTCCGTTGTCTCC  R3: TCCGTGCAGACAGGGTTC | 60°C, 35 cycles | N/A |
| 21 | 37 bp inside Exon 35 | 34 bp inside Exon 41 | F:  GACAACGGAAACGCTGACG  R:  GTCGTACACAGCGCAGTAGC | F2: ATGATACCCTGGTGGAGAGC  F3: ACAGAGTGCCATCAAGCAGG  R2: CCAAGGAGTGAGCGTGAC  R3: GCGAAGACTCCGATTCCTTAG | 60°C, 35 cycles | As for fragment 1 |
| 22 | 100 bp inside Exon 40 | 94 bp downstream of transcription termination code | First round:  F:  CTTCAGCACCGGGGACTAC  R:  ACGAGGCTGCCTACTCCG  Second round  F:  ATTGGCTGGGGTAGTGCTGC  R:  ACCTTGAGGGCAGCAGAAG | F2: GTTCCCCATGCGTTACATTC  F3: CTCTTCGCGCTGTACTTCTC  F4: TCGCTGCTCTTCCTGCTC  R2: CTCCACTCAGCGTGTCCAG  R3: GCCGGTCGGTAGAGTTCC  R4: GTGGACAAGTGGGGTCAG | First round:  62°C, 35 cycles  Second round:  62°C, 28 cycles | Annealing temperature of 60°C, cyclical denaturation of 96°C for 30s |

^1^The number of nucleotide residues from the 5’ ends of the predicted UTR, intron or exon

^2^bp; Base pair

^3^F; Forward primer

^4^R; Reverse primer

^5^Variations in annealing temperatures and number of cycles used for amplifying each PCR fragment, compared to the standard PCR conditions (refer to the text for standard conditions)

^6^Variations in conditions used for sequencing of different PCR fragments, compared to the standard sequencing conditions (refer to the text for standard conditions)

^7^N/A; Not applicable
